# MEG masked priming evidence for form-based decomposition of irregular verbs

**DOI:** 10.3389/fnhum.2013.00798

**Published:** 2013-11-22

**Authors:** Joseph Fruchter, Linnaea Stockall, Alec Marantz

**Affiliations:** ^1^Department of Psychology, New York UniversityNew York, NY, USA; ^2^Department of Linguistics, Queen Mary, University of LondonLondon, UK; ^3^Department of Linguistics, New York UniversityNew York, NY, USA; ^4^NYUAD Institute, New York University Abu DhabiAbu Dhabi, UAE

**Keywords:** masked priming, MEG and EEG, neurolinguistics, visual word recognition, morphological processing, past tense debate

## Abstract

To what extent does morphological structure play a role in early processing of visually presented English past tense verbs? Previous masked priming studies have demonstrated effects of obligatory form-based decomposition for genuinely affixed words (teacher-TEACH) and pseudo-affixed words (corner-CORN), but not for orthographic controls (brothel-BROTH). Additionally, MEG single word reading studies have demonstrated that the transition probability from stem to affix (in genuinely affixed words) modulates an early evoked response known as the M170; parallel findings have been shown for the transition probability from stem to pseudo-affix (in pseudo-affixed words). Here, utilizing the M170 as a neural index of visual form-based morphological decomposition, we ask whether the M170 demonstrates masked morphological priming effects for irregular past tense verbs (following a previous study which obtained behavioral masked priming effects for irregulars). Dual mechanism theories of the English past tense predict a rule-based decomposition for regulars but not for irregulars, while certain single mechanism theories predict rule-based decomposition even for irregulars. MEG data was recorded for 16 subjects performing a visual masked priming lexical decision task. Using a functional region of interest (fROI) defined on the basis of repetition priming and regular morphological priming effects within the left fusiform and inferior temporal regions, we found that activity in this fROI was modulated by the masked priming manipulation for irregular verbs, during the time window of the M170. We also found effects of the scores generated by the learning model of Albright and Hayes (2003) on the degree of priming for irregular verbs. The results favor a single mechanism account of the English past tense, in which even irregulars are decomposed into stems and affixes prior to lexical access, as opposed to a dual mechanism model, in which irregulars are recognized as whole forms.

## Introduction

### Background: past tense debate

The distinction between regular (e.g., *jump*-*jumped*) and irregular (e.g., *teach*-*taught*) morphology in the English past tense has served as the basis for much debate in the psycholinguistic literature. Some have argued for a dual mechanism account, in which regular verbs are generated from their stems by rule, and irregular verbs are memorized as whole forms and stored in the lexicon (Pinker and Prince, 1988; Pinker, 1991). Under this account, irregulars, hypothesized to be stored as whole forms, are predicted to display surface (i.e., whole word) frequency effects, while regulars, hypothesized to be computed by rule from a stem and suffix, are predicted to display stem frequency effects; Pinker (1991) cites confirmatory evidence from experiments on ratings of past tense forms, as well as reaction times (RTs) in a verb generation task. Similarly, separate neural bases for regular and irregular inflection are also predicted by this account. Utilizing an ERP morphological violation paradigm, Luck et al. (2006) found that auditory presentation of invalid words generated by adding a regular suffix to a stem that requires irregular suffixation (i.e., overregularizations) elicited LAN/P600 effects, while presentation of invalid words generated by adding an irregular ending to a stem that requires regular suffixation (i.e., irregularizations) produced N400 effects. These findings were interpreted as illustrating the syntactic nature of overregularization, since the LAN and P600 are generally associated with syntactic violations (Friederici, 2002), and the lexical nature of irregularization, since the N400 is generally associated with word-level violations (Kutas and Schmitt, 2003). In an fMRI experiment, Vannest et al. (2005) compared suffixed words that showed behavioral evidence of decomposition (i.e., stem frequency effects for words ending in -*ness*, -*less*, and -*able*) with suffixed words that failed to show such effects (i.e., only surface frequency effects for words ending in-*ity* and -*ation*), and they found that decomposability was associated with increased levels of activity in Broca's area and the basal ganglia (argued by Ullman et al., 1997, to be part of the procedural circuit for grammatical rule processing). The distinction between surface frequency and stem frequency effects was thus taken by Vannest et al. (2005) as a diagnostic for the distinction between storage and computation, allowing them to argue for separate neural bases for the processing of decomposable and non-decomposable complex words.

Some have argued for alternative models of the English past tense, in which regular and irregular inflection are processed by a single mechanism. Under one such theory, regular and irregular verbs are both represented in a single connectionist network with quantifiable mappings between stems and candidate past tense forms (Rumelhart and McClelland, 1986; see McClelland and Patterson, 2002a,b for specific arguments in favor of single mechanism connectionist models of morphological complexity and irregularity). A different type of single mechanism account is advanced by Stockall and Marantz (2006), who argue that both regular and irregular verbs are composed by rule from their stems, in contrast both to the connectionist account of morphological relationships as a type of similarity, and to the dual mechanism account in which only regulars are composed by rule from their stems. Their evidence for this position comes from an MEG evoked response associated with lexical access (the M350) that displayed equivalent morphological priming effects for both regular and irregular verb-stem pairs, but no priming effects for pairs such as *boil-broil*, which are phonologically and semantically similar, but have no plausible morphological relationship.

It should be noted that under both types of single mechanism account, frequencies associated with the computation of the past tense should be relevant during the early stages of recognition of past tense verbs, for both regulars and irregulars; in contrast, under the dual mechanism account, only surface frequency should be relevant during the early stages of recognition of irregular verbs, and thus the results of Pinker (1991) and Vannest et al. (2005), inter alia, would seem to argue against such single mechanism models.

However, in the recent psycholinguistic literature, there have been findings that complicate the previously drawn binary distinction between storage and computation, as measured by the difference between surface frequency and stem frequency effects. Taft (2004) noted that stem frequency effects may be attenuated by the later stage of recombination of stem and affix: specifically, when matched for surface frequency, complex words with higher stem frequencies are more difficult to recombine than those with lower stem frequencies, thus canceling out the earlier stem recognition advantage. Baayen et al. (2007) argued that the dichotomy between storage and computation is false, since even low frequency regular verbs show effects of being stored in memory (i.e., surface frequency effects). Albright and Hayes (2003) presented behavioral ratings data for novel past tense forms, which demonstrated an influence of the phonological features of the stem, for both regulars and irregulars. One conclusion that we can draw from these findings is that processing of regular verbs is affected by a language user's prior experience with productive combination of their constituent morphemes. In other words, regulars are similar to irregulars, in that they display the effects of experience with their complex forms. In the present study, we ask the converse question: are irregulars similar to regulars, in that they show the effects of decomposition into their constituent morphemes?

If the predictions of the single mechanism account of Stockall and Marantz (2006) are correct, then we should find evidence for early visual word form based decomposition of irregular verbs. In order to experimentally verify these predictions, we combine MEG recordings with the behavioral masked priming paradigm (Forster and Davis, 1984). We contrast the predictions of Stockall and Marantz (2006) with those of the dual mechanism theory, in which irregulars are not predicted to be decomposed into stems and affixes at the early stages of word recognition. We do not specifically test the predictions of the single mechanism connectionist account, since any such predictions would be highly dependent on the details of a particular instantiation of a connectionist network (see Seidenberg and Plaut, in press for a discussion of the issues involved in taking any particular connectionist model as representative or complete).

### Early stages of visual processing of complex regular words

There is much evidence for the importance of morphological structure during the early stages of visual word recognition. Rastle et al. (2004) reported findings from a masked priming experiment, which demonstrated significant levels of RT priming for genuinely affixed word-stem pairs (e.g., teacher-TEACH), as well as for pseudo-affixed word-stem pairs (e.g., corner-CORN), but not for pairs exhibiting similar orthographic overlap, but which cannot be exhaustively parsed into a possible stem and affix (e.g., brothel-BROTH; see Rastle and Davis, 2008 for a review of 19 studies reporting similar findings). Since real affixation and pseudo-affixation (but not simple orthographic overlap) lead to masked priming effects, we can conclude that the visual word recognition system is sensitive to the potential presence of morphological structure, before the lexical representation of a word is accessed; the latter point follows from the fact that pseudo-affixed words would presumably not be decomposed if the lexical entry were already retrieved, and the apparent morphological decomposition found to be erroneous. Thus, findings from the psycholinguistic literature can be taken as support for the presence of an early stage of morphological decomposition of visual word forms, independent of semantics, which takes place prior to lexical access.

There is also neural evidence, from non-priming paradigms, for early form-based morphological decomposition of complex visual words. MEG studies of visual word recognition, employing a single word reading paradigm and using correlational analyses to model evoked neural effects, have shown that an early evoked response from the visual word form area (Cohen et al., 2000) in the left fusiform gyrus, known as the M170 (Pylkkänen et al., 2002), is modulated by the transition probability from stem to affix in derivationally complex words [e.g., *p*(*teacher* |*teach*); Solomyak and Marantz, 2010], as well as the transition probability from (pseudo-)stem to (pseudo-)affix in pseudo-affixed words [e.g., *p*(*corner* |*corn*); Lewis et al., 2011]. Thus, the M170 can be regarded as a neural index of visual morphological decomposition, insofar as it is sensitive to statistical variables related to the morphological structure of visual word forms. However, previous M170 results have only involved derivational morphology; it thus remains an open question as to whether inflectional morphology will play a similar role in modulating the M170. If the M170 does indeed show sensitivity to inflectional morphology, then it is reasonable to predict that it should be modulated by a masked priming manipulation with past tense verbs.

In summary, there is strong, convergent support for visual word form based morphological decomposition, which occurs rapidly and automatically for all potentially complex regular words. This decomposition seems blind to semantic factors, though sensitive to transitional probabilities of the component morphemes. In the present study, we will utilize the neural index of the decomposition process (i.e., the M170) to investigate processing of inflectional morphology. Given this background for the behavioral and neural consequences of regular affixal morphological decomposition, we can now turn specifically to the issue of irregular past tense morphology.

### Modeling irregular past tense morphology

Albright and Hayes (2003) conducted a computational test of a single mechanism account of the English past tense, which featured stochastic rules as the basis of past tense generation^1^. The evidence for their rule-based account consisted of behavioral ratings of novel past tense forms; crucially, ratings of both regular and irregular forms were affected by the phonological features of their respective stems, suggesting raters were making use of these stem features in evaluating the well-formedness of the inflected forms. For both Albright and Hayes (2003) and Stockall and Marantz (2006), irregular inflection is generated by rule, such that, for example, one might produce *gled* as the past tense form of the novel verb *gleed*, due to the morphophonological rule i→ε/[X{l,r}_d][+past] (as in *lead-led*, *bleed-bled, breed-bred*, etc.). Albright and Hayes (2003) refer to a phonological context of relatively high consistency for a particular rule as an “island of reliability.” Interestingly, their results demonstrated that English speakers were sensitive to such islands of reliability not only for novel irregular verbs (e.g., *fleep-flept* and *gleed-gled*), but also for novel regular verbs (e.g., *bredge-bredged* and *nace-naced*). The latter finding is contrary to the predictions of the dual mechanism theory, in which all regular verbs are derived via a single rule (with three predictable allomorphs: -*t*, -*d*, -*∂d*), and thus would not be expected to demonstrate effects of differing phonological contexts.

Albright and Hayes (2003) also developed a computational model that learned rules for the mapping from the phonological form of a stem to the phonological form of the past tense. Using this model, we were able to derive scores for the past tense verbs in our study, which represent the degree to which a particular past tense verb is supported by the morphophonological rules governing past tense formation in general (this measure will be referred to as “AlbrightScore”). Within the irregular verbs, there was a wide distribution over the AlbrightScore measure (from a minimum value of 0 to a maximum value of 1): for example, the irregular pair *send*-*sent* has a relatively high value for AlbrightScore (0.72), since it was supported by the related pairs *lend*-*lent* and *rend*-*rent*, while the irregular pair *fly*-*flew* had the lowest possible value for AlbrightScore (0), since it was not supported by any related forms.

We tested the effect of AlbrightScore on the level of M170 morphological priming, in order to look for evidence of rule application during processing of irregular verbs: specifically, we predicted that irregular morphological priming effects would be stronger for those verbs with higher AlbrightScore (e.g., *send*-*sent*), since they would have a greater degree of support for their particular past tense inflections from the overall rule structure governing past tense formation. Such evidence would support a rule-based decomposition model for all past tense verbs, as argued for by Stockall and Marantz (2006); it would also be contrary to the predictions of the dual mechanism account, under which irregular verbs (such as *sent*) are retrieved as whole forms from the lexicon.

### Behavioral masked priming: evidence for decomposition of irregulars

As outlined above, given the extensive theoretical debate regarding the English past tense, we designed the present study to investigate the following question: does early form-based decomposition take place only on the basis of regular affixal morphology, or does it also apply to irregular morphology? Since stem allomorphy is extremely pervasive and systematic across languages, it seems highly unlikely that a sophisticated visual linguistic pattern detection system would only be capable of detecting affixal morphology. In fact, a recent behavioral study has shown that, despite the lack of a visual morphemic segmentation for irregularly inflected words, masked presentation of such words facilitated lexical decision RTs to their corresponding stems, more than orthographically related primes and unrelated control primes (Crepaldi et al., 2010). This study also included a pseudo-irregular condition containing words that shared the orthographic sub-regularities of the irregular items (e.g., bell-BALL matches the orthographic pattern in fell-FALL). If the masked irregular priming effects are due to an early visual word form based decomposition using these orthographic sub-regularities, then the pseudo-irregular condition would be predicted to show the same masked priming effects. Contrary to this prediction, Crepaldi et al. (2010) found no such pseudo-irregular priming effect. Since this finding seems to argue against a form-based decomposition mechanism operating over irregularly inflected forms, they interpret the result as implying the existence of an additional lemma level source of morphological priming. However, their conclusion may be premature, since they matched their pseudo-irregular items to real irregular items based only on their orthographic patterns, while allowing divergence in their phonological patterns (e.g., drought-DRINK was matched to thought-THINK). We matched our pseudo-irregulars to real irregulars based on orthography as well as phonology^2^.

Despite this complication with the pseudo-irregular condition, the behavioral masked priming evidence is consistent with a single mechanism account of the past tense: complex words seem to be decomposed into their stems, irrespective of whether they contain regular or irregular morphology. Since effects of semantic relatedness are not typically observed in a masked priming paradigm (at least for an SOA of 43 ms; Rastle et al., 2000), the priming observed for even irregular verbs must be form-based: brief (i.e., <50 ms) exposure to the irregular past tense form *left* is sufficient to parse this form as *lef* + *-t*, and to recognize *lef* as an allomorph of *leave*. It is less obvious how to explain irregular decomposition under a dual mechanism theory, in which the nature of the connection between irregular verbs and their stems is that of a semantic link between different lexical items.

### MEG/EEG priming literature

Though there have been several previous MEG studies of visual word priming, none of these studies have presented clear data relating the priming manipulation to the M170. Stockall and Marantz (2006) utilized an overt (i.e., unmasked) priming paradigm, which provided evidence that both regular and irregular verbs prime their stems; however, their dependent measure was the M350, a late evoked response from the left superior and middle temporal regions that has been associated with lexical access (Pylkkänen and Marantz, 2003).

A number of recent studies have combined masked morphological priming with EEG or MEG measurements, but the earliest evoked response showing sensitivity to morphological complexity peaks between 200 and 300 ms (EEG: Lavric et al., 2007; Morris et al., 2007, 2008; Morris and Stockall, 2012; Royle et al., 2012; MEG: Lehtonen et al., 2011). Lavric et al. (2007), Morris et al. (2008), and Morris and Stockall (2012), all using EEG to measure neural processing, do report sensitivity to masked repetition priming in an evoked response peaking 130–200 ms after target onset (N/P 150), but Monahan et al. (2008), using MEG, find the earliest effects of masked repetition priming at ~225 ms. The EEG studies also report a later masked priming effect, namely an attenuation of the N400 response, which is sensitive to both repetition priming and morphological priming (Lavric et al., 2007; Morris et al., 2008; Morris and Stockall, 2012).

Given that there is evidence that the lexical access process has already begun at 300 ms (or earlier), from an MEG study of homonyms that demonstrated effects of meaning entropy at this latency (Simon et al., 2012), and given the MEG single word reading evidence for sensitivity to morphological complexity in the M170 response, the lack of any observed M170 masked priming sensitivity is surprising.

A preliminary goal of our study is thus to investigate whether there is indeed an M170 masked priming effect, in general. An important difference between the current study and the previous EEG and MEG masked priming research is that rather than analyze averaged sensor data, we use minimum norm estimation to determine the plausible neural generators of the evoked sensor data, and then use anatomically and functionally defined regions of interest (ROIs) to constrain our analyses in source space. As outlined above, a further goal of our study is to investigate whether there is an early form-based masked morphological priming effect for irregular verbs specifically. Such an effect would be consistent with the single mechanism account of the English past tense (Stockall and Marantz, 2006), as well as with the behavioral masked priming results (Crepaldi et al., 2010). It would also highlight the importance of the M170 as an index of visual form-based morphological decomposition, not only for the previously studied cases of regular derivational morphology, but also for inflectional morphology, both regular and irregular. Finally, given the EEG evidence for N400 effects of masked priming, we also verify that there is a later MEG effect of masked priming, during the time window of the M350/N400m (i.e., the MEG evoked response analogous to the N400, discussed in Helenius et al., 1998 and Halgren et al., 2002).

## Materials and methods

### Design and stimuli

Our experiment consisted of a visual masked priming lexical decision task, with simultaneous MEG recording of the magnetic fields induced by electrical activity in the brain. There were four conditions of interest, with 50 trials in each condition: **identity** (car-CAR), **regular** (jumped-JUMP), **irregular** (fell-FALL), and **pseudo-irregular** (bell-BALL). The irregular and pseudo-irregular items were matched on both their orthographic and phonological patterns. Primes were presented in lower case and targets were presented in upper case, in order to ensure that any priming effects would not be due merely to repetition of the low-level visual features of the stimuli. There were an equal number of trials in which the same targets were preceded by unrelated primes (wing-FALL). We did not include orthographic or semantic control conditions, since there is no evidence of a facilitatory masked priming effect for orthographically or semantically similar words, given the SOA (33.3 ms) and the average word length (4.2 letters) of the stimuli in this experiment^3^. Words were excluded from our study if they had a mean accuracy rate below 55% in lexical decision tasks, as measured by the English Lexicon Project, or ELP (Balota et al., 2007).

Frequency counts for the words in this experiment were obtained from CELEX (Baayen et al., 1995). Surface frequency for the regular and irregular verb primes was taken to be the logarithm of the CELEX wordform frequency for the particular past tense verb.

Table 1 summarizes the mean values of word length, log surface frequency, and orthographic neighborhood size for the different experimental conditions. We chose the primes for these conditions so as to minimize the difference between related and unrelated primes along the above three dimensions; in particular, the related and unrelated primes were pairwise matched for word length, and listwise matched for surface frequency and orthographic neighborhood size, for each of the different conditions of interest (identity, regular, irregular, and pseudo-irregular).

**Table 1:** **Mean values of word length, log CELEX surface frequency (Freq), and orthographic neighborhood size (*N*) for the different experimental conditions**.

**Condition**	**Type**	**Word length**	**Freq**	***N***
Identity	Target	5.68 ± 1.04	2.60 ± 0.51	3.00 ± 4.36
	Related prime	5.68 ± 1.04	2.60 ± 0.51	3.00 ± 4.36
	Unrelated prime	5.68 ± 1.04	2.63 ± 0.58	2.82 ± 3.86
Regular	Target	4.14 ± 0.45	3.06 ± 0.55	9.30 ± 3.65
	Related prime	6.12 ± 0.44	2.88 ± 0.50	4.96 ± 2.43
	Unrelated prime	6.12 ± 0.44	2.75 ± 0.50	4.72 ± 2.42
Irregular	Target	4.20 ± 0.76	2.80 ± 0.72	8.92 ± 5.13
	Related prime	4.20 ± 0.83	2.76 ± 0.68	8.40 ± 4.89
	Unrelated prime	4.20 ± 0.83	2.71 ± 0.71	8.12 ± 4.65
Pseudo-irregular	Target	3.88 ± 0.66	2.50 ± 1.00	11.74 ± 4.78
	Related prime	3.88 ± 0.82	2.26 ± 0.88	11.82 ± 4.92
	Unrelated prime	3.88 ± 0.82	2.31 ± 0.83	11.32 ± 4.58
Total word	Target	4.48 ± 1.03	2.74 ± 0.75	8.24 ± 5.52
	Related prime	4.97 ± 1.25	2.62 ± 0.70	7.05 ± 5.42
	Unrelated prime	4.97 ± 1.25	2.60 ± 0.68	6.75 ± 5.12
	Total prime	4.97 ± 1.25	2.61 ± 0.69	6.90 ± 5.27
Total non-word	Target	4.48 ± 1.03	N/A	8.03 ± 5.35
	Related prime	4.11 ± 0.71	2.57 ± 0.69	8.60 ± 4.87
	Unrelated prime	5.17 ± 1.23	2.52 ± 0.68	6.03 ± 5.44
	Total prime	4.77 ± 1.19	2.54 ± 0.68	7.00 ± 5.37

We also selected 200 non-word targets from the ELP, which could be transformed into real words upon substitution of a single letter. We sought to minimize the difference between the mean values of word length and orthographic neighborhood size for the word and non-word targets; the non-word targets were thus pairwise matched to the word targets for length, and they were listwise matched for neighborhood size. The non-word targets were preceded by real word primes: 75 were orthographically related via the single letter substitution, and 125 were orthographically unrelated. Of the unrelated primes, 25 ended in “-ed” in order to match the 25 related primes in the regular verb condition. The primes for the non-word targets were listwise matched to the primes for the word targets on all three variables. None of the primes were non-words, in order to ensure that the lexicality of the prime could not be used as evidence toward the lexical decision on the target. The stimuli for this experiment are listed in the Appendix.

Since we did not want a given participant to view the same target twice, we developed two versions (A and B) of the experiment. In each version, half of the real word targets were preceded by related primes, and the other half were preceded by unrelated primes; the 200 non-word trials remained the same in both versions of the experiment. Versions A and B were counterbalanced across participants. Thus, a given participant viewed a total of 200 unique real word targets (preceded by 100 related primes and 100 unrelated primes, from version A or B) and 200 unique non-word targets (preceded by 75 related primes and 125 unrelated primes, with no difference between versions).

AlbrightScore values were generated for the regular and irregular verbs, using the past tense learner program available online (Albright, 2003). The input to the learner consisted of the phonological representations of the verb stems in our experiment. The score for a given past tense form was taken from the program's output, if available for that verb; otherwise, if the program did not produce a given inflection, the AlbrightScore was assigned to be 0 (i.e., the minimum value for the measure). The AlbrightScore measure thus ranged from 0 (no support for the past tense form) to 1 (complete support for the past tense form).

### Experimental procedures

Sixteen right-handed native English speakers (8 males and 8 females) participated in the MEG experiment. All subjects provided written informed consent to participate in the study.

DMDX (Forster and Forster, 2003) was used as the presentation platform for the experiment. The font was Courier New, size 28. Each trial of the experiment consisted of a string of hash marks appearing for 500 ms (“#######”), a lower-case prime appearing for 33.3 ms (“fell”), and an upper-case target displayed for 300 ms (“FALL”). Subjects were instructed to respond to the target stimulus by pressing one button if they recognized the string as a valid word of English, and a second button if the string was invalid. After the experiment, subjects were asked whether they were able to read the masked primes; none of the subjects indicated an ability to do so.

A 157-channel axial gradiometer whole-head MEG system (Kanazawa Institute of Technology, Kanazawa, Japan) recorded the MEG data at a sampling frequency of 1000 Hz. The data was filtered between DC and 500 Hz, with a band elimination filter of 60 Hz. The subjects' heads were digitized prior to entering the magnetically shielded room. The head positions during the experiment were determined via coils attached to anatomical landmarks. Structural MRIs were also obtained for all the subjects, and the coil locations were used to translate from the MEG spatial coordinates to the MRI coordinates.

### Analysis

#### Behavioral analysis

Reaction times and accuracy data were recorded for each trial of the lexical decision task. Subjects with a mean RT greater than 2 standard deviations above the mean RT for all subjects, or an RT standard deviation greater than 2 standard deviations above the mean RT standard deviation for all subjects, were removed from the behavioral analysis; this resulted in the removal of two subjects, while maintaining the counterbalancing between the two versions of the experiment. Trials with an RT that was either less than 300 ms or greater than 2 standard deviations above the mean RT across subjects (within the given condition) were also removed from the behavioral analysis. Two of the subjects had accuracy rates slightly worse than 2 standard deviations below the mean accuracy rate (88.75 and 89%), but we included them in the analysis, since removing their data would ruin the counterbalancing across the two versions of the experiment.

In order to analyze the correlation of RT with the masked priming manipulation, we used linear mixed effects models (Baayen et al., 2008) with RT as the dependent variable, PrimeType (related vs. unrelated) as the fixed effect, and subject and item as random effects. The linear mixed effects models were constructed using the lmer function of the lme4 package in R (Bates and Maechler, 2009). The *p*-values were computed via Monte Carlo (MC) simulation with 10,000 iterations each. In order to determine whether the pseudo-irregular items displayed a significantly different level of priming than the irregular items, following Crepaldi et al. (2010), we analyzed the interaction between PrimeType and Pseudo-irregularity (i.e., irregular vs. pseudo-irregular) for the irregular and pseudo-irregular items only. In order to analyze this interaction, we first fit a linear mixed effects model with PrimeType and Pseudo-irregularity as fixed effects. We then fit a second linear mixed effects model with the two measures and their interaction as fixed effects. Finally, we performed a likelihood ratio test of the two nested models, which produces a χ^2^-value and an associated *p*-value, indicating the significance of adding the interaction term to the model.

#### MEG analysis

***Data analysis***. The MEG data was noise reduced via the Continuously Adjusted Least-Squares Method (Adachi et al., 2001), in the MEG160 software (Yokogawa Electric Corporation and Eagle Technology Corporation, Tokyo, Japan). Cortically constrained minimum-norm estimates were calculated via MNE (MGH/HMS/MIT Athinoula A. Martinos Center for Biomedical Imaging, Charleston, MA). The cortical reconstructions were obtained using FreeSurfer (CorTechs Labs Inc., La Jolla, CA and MGH/HMS/MIT Athinoula A. Martinos Center for Biomedical Imaging, Charleston, MA). A source space of 5124 points was generated for each reconstructed surface, and the BEM (boundary-element model) method was employed on activity at each source to calculate the forward solution. Using the grand average of all trials for a particular subject, after baseline correction with the pre-target interval (−150, −50 ms) [or, equivalently, the interval (−117, −17 ms) relative to the presentation of the prime] and low pass filtering at 40 Hz, the inverse solution was computed from the forward solution, in order to determine the most likely distribution of neural activity. The inverse solution was computed with a free orientation for the source estimates, meaning that the estimates were unconstrained with respect to the cortical surface. The resulting minimum norm estimates were signed, with positive values indicating an upward directionality, and the negative values indicating a downward directionality, in the coordinate space defined by the head^4^. The signed estimates were transformed into (signed) noise-normalized dynamic statistical parameter maps (dSPMs; following Dale et al., 2000). FreeSurfer's automatically-parcellated anatomical ROIs were used to obtain estimates of the average noise-normalized neural activity (i.e., dSPM values) within left temporal cortical regions. In order to analyze the grand-averaged evoked activity across all subjects, we morphed each individual subject's brain to the common space of a single representative subject's brain. In order to analyze the functionally defined ROI (fROI), we drew an ROI in the common neuroanatomical space, morphed it back into each individual subject's neuroanatomical space, and extracted the average dSPM values within the fROI for each subject.

Outlier trials were removed based on an absolute threshold of ±2.5 pT, enforced over the time window (−150, +300 ms) for the noise reduced MEG data.

**Figure 1:**
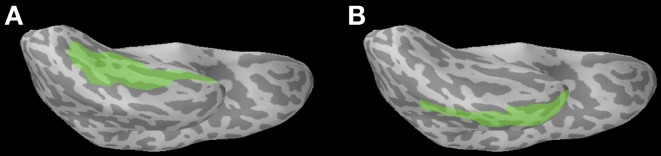
**Location of anatomical ROIs, highlighted in green on a representative subject's inflated cortical surface (ventral view, left hemisphere): (A)** The fusiform ROI (used for the M170 analysis), and **(B)** The middle temporal ROI (used for the M350/N400m analysis).

***Anatomical ROI analysis***. We examined two cortical areas of interest within the left temporal lobe, since this general location is associated with the M170 response and the M350 response (Pylkkänen and Marantz, 2003; Solomyak and Marantz, 2010). In particular, we used the FreeSurfer-generated anatomical ROIs for the fusiform and middle temporal regions (Figure 1).

For the M170 analysis, we investigated the effect of PrimeType (related vs. unrelated) on activity in the fusiform ROI; the time window of interest was a 50 ms interval centered at the peak of the M170 (i.e., the peak of the mean fusiform activity across trials and across subjects). For the M350/N400m analysis, we investigated the effect of PrimeType on activity in the middle temporal ROI; the time window of interest was the general late interval 300–500 ms post-target onset.

**Figure 2:**
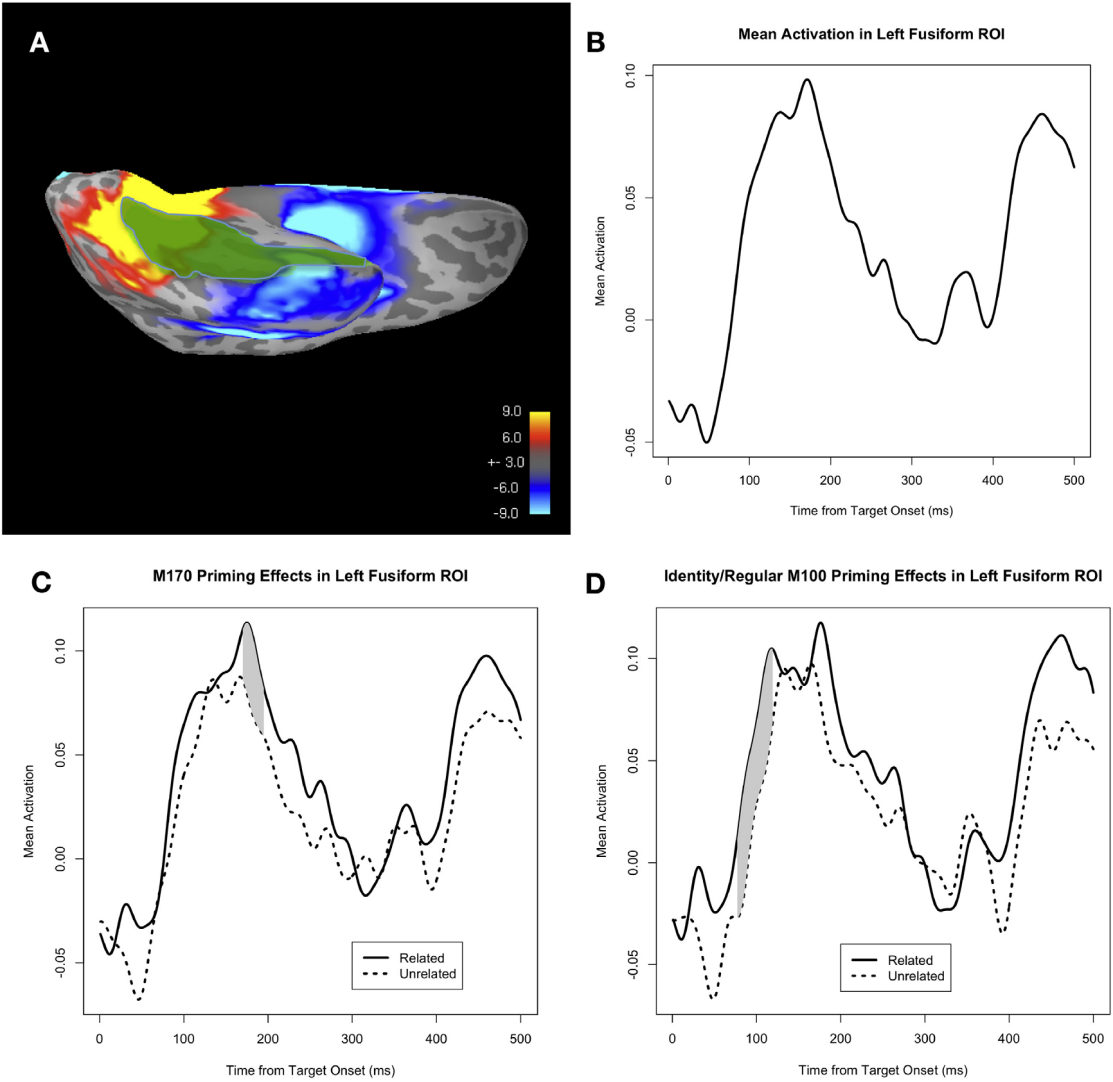
**(A)** Mean whole-brain activity across all subjects and all trials at 170 ms post-target onset, shown on a representative subject's inflated cortical surface (ventral view, left hemisphere). Positive activity (i.e., upward with respect to the head) is shown in red/yellow, and negative activity (i.e., downward with respect to the head) is shown in blue. The anatomical fusiform ROI is highlighted in green. **(B)** Mean activity in the fusiform ROI, collapsed across all four conditions: identity, regular, irregular, and pseudo-irregular. **(C)** Mean activity in the fusiform ROI, separated by PrimeType, and pooled across the 4 conditions of identity, regular, irregular, and pseudo-irregular. The solid line represents the related PrimeType condition, and the dashed line represents the unrelated PrimeType condition. The significant M170 priming effect is shaded in gray; note that other time windows were not tested for significance. **(D)** Mean activity in the fusiform ROI, separated by PrimeType, and pooled across the identity and regular conditions. The solid line represents the related PrimeType condition, and the dashed line represents the unrelated PrimeType condition. The significant M100 priming effect is shaded in gray; note that other time windows were not tested for significance.

***Functional ROI analysis***. In our analysis of the grand-averaged evoked activity across all subjects and all trials in the experiment (Figure 2A), we observed a large patch of positive (i.e., upward) activity in the occipitotemporal region, as well as a separate patch of negative (i.e., downward) activity more anteriorly within the temporal lobe. Both of these patches of activity overlapped with the fusiform ROI; the former positive patch overlapped with the posterior part of the fusiform, and the latter negative patch overlapped with the anterior part of the fusiform. The time course of the positive patch was consistent with the M170 response, showing a positive peak at ~170 ms post-target onset, while the time course of the negative patch showed a more gradual decline in the negative (downward) direction (not shown). The presence of two separate response components within the same ROI yields a potential confound for our anatomical ROI analysis of the M170 priming effect. Due to the uncertainty arising from this confusion of separate evoked responses, we decided to conduct a functional region of interest (fROI) analysis as well.

**Figure 3:**
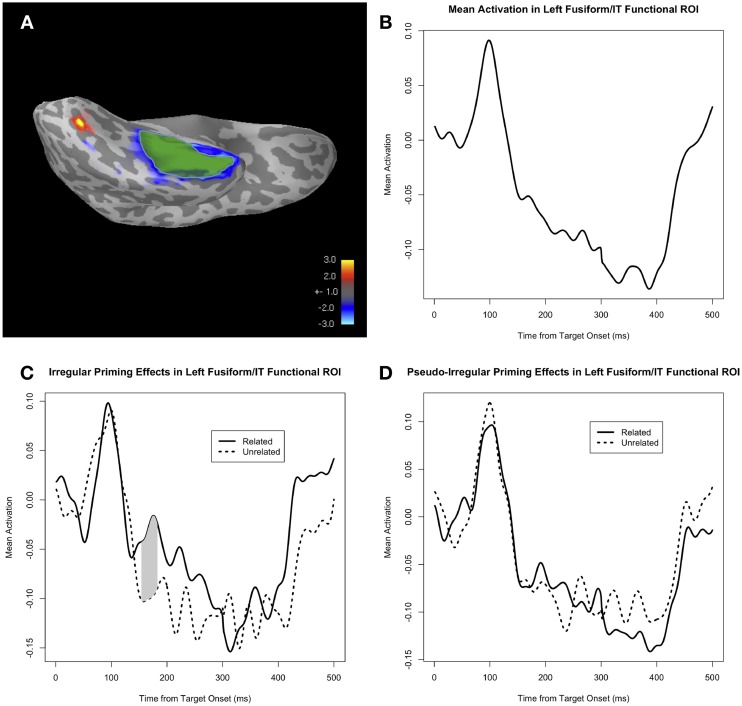
**(A)** The priming effect across all subjects, collapsed across all trials in the identity and regular conditions, shown on a representative subject's inflated cortical surface, at approximately the temporal peak of the effect (i.e., ~190 ms post-target onset). Facilitatory priming effects are shown in blue, and inhibitory priming effects are shown in red. The functionally defined ROI (fROI) for the facilitatory priming effect, within the fusiform and inferior temporal ROIs, is highlighted in green (overlaid on the blue patch representing the facilitatory priming effect). **(B)** Mean activity in the fROI, collapsed across all four conditions: identity, regular, irregular, and pseudo-irregular. **(C)** Mean activity in the fROI, separated by PrimeType, for the irregular condition. The significant M170 priming effect is shaded in gray; note that other time windows were not tested for significance. **(D)** Mean activity in the fROI, separated by PrimeType, for the pseudo-irregular condition.

We defined an fROI on the basis of the identity and regular priming conditions (i.e., repetition priming and regular morphological priming). Specifically, within the cortical area covered by the fusiform and inferior temporal anatomical ROIs in the common neuroanatomical space of a representative subject's brain, we drew an fROI around the peak facilitatory priming effect^5^ (across all subjects) in the identity and regular conditions combined, during the time window around the M170 (Figure 3A). We then morphed this fROI from the representative subject's brain to the neuroanatomical space for each individual subject. We investigated the effect of PrimeType (related vs. unrelated) on activity within this fROI for the irregular and pseudo-irregular conditions. Additionally, we investigated whether there was an interaction of AlbrightScore and PrimeType for the irregular verbs; specifically, we hypothesized that there would be a greater priming effect for the irregular verbs that had a higher AlbrightScore value.

***Statistical methodology***. To analyze the masked priming effects in the MEG data, we employed linear mixed effects models (Baayen et al., 2008) millisecond-by-millisecond (i.e., we used separate models at each time point), with the average neural activity in an ROI as the dependent variable, PrimeType as the fixed effect, and subject and item as random effects. The *t*-values for the fixed effect^6^ were then corrected for multiple comparisons over the selected time window of interest only. The linear mixed effects models were constructed using the lmer function of the lme4 package in R (Bates and Maechler, 2009). The technique that we used for multiple comparisons correction is based on the methods of Maris and Oostenveld (2007), as adapted by Solomyak and Marantz (2009). Specifically, we computed Σt, the sum of all *t-values* within a single temporal cluster of consecutive significant effects in the same direction (where significant is defined by |*t*| > 1.96, *p* < 0.05 uncorrected). The highest absolute value of Σt, for any cluster within the whole time window, was then compared to the results of the same procedure repeated on 10,000 random permutations of the independent variable (i.e., PrimeType). An MC *p*-value was thus computed, based on the percentage of times a random permutation of the independent variable led to a larger maximum absolute value of Σt than the original maximum absolute value of Σt (as computed on the actual data).

In order to analyze interaction effects, say for measures A and B, we first fit a linear mixed effects model with A and B as fixed effects. We then fit a second linear mixed effects model with A, B, and their interaction as fixed effects. Finally, we performed a likelihood ratio test of the two nested models, which produces a χ^2^-value, indicating the significance of adding the interaction term to the model. To correct for multiple comparisons over a time window of interest, we performed a similar procedure to the one described above, except with the square root of the χ^2^-values rather than *t*-values, and with random permutations of two independent variables (A and B).

#### Albrightscore analysis

We also conducted a test of the scores generated by the past tense learning model from Albright and Hayes (2003). More specifically, we analyzed the interaction of AlbrightScore with PrimeType for the irregular verbs, in order to test whether the gradient measure of a past tense form's support from the various past tense phonological rules (i.e., its AlbrightScore) would impact the degree of priming from the masked past tense form to its corresponding stem. We performed this analysis for both the behavioral data (i.e., RT) and the MEG data (i.e., the M170 response), using the same methodology described in the above sections. Due to the extremely high mean AlbrightScore for the regular verbs (close to the maximum possible value, in fact), we refrained from performing a comparable AlbrightScore analysis for those items.

## Results

### Behavioral results

The mean accuracy rate across all subjects was 94.4% (±2.65%). The mean RT across all subjects was 620.7 ms (±178.7 ms). Significant RT priming was found for the identity condition (33.3 ms; *t* = 4.66, MC-corrected *p* = 0.0001), the regular condition (22.5 ms; *t* = 3.21, MC-corrected *p* = 0.002), and the irregular condition (14.2 ms; *t* = 2.07, MC-corrected *p* = 0.042). The pseudo-irregular condition displayed a trend toward significance (14.6 ms; *t* = 1.72, MC-corrected *p* = 0.083). Our behavioral analysis showed no significant interaction between PrimeType and Pseudo-irregularity (χ^2^ = 0.002, *p* = 0.97), consistent with the fact that the irregulars and pseudo-irregulars demonstrated comparable levels of priming.

### MEG results

Visual inspection of the grand-averaged evoked fusiform activity (Figure 2B) reveals that it peaks in the positive direction (i.e., upward with respect to the head) during the time window 100–200 ms post-target onset. In fact, there appears to be an earlier peak at 133 ms and a later peak at 171 ms; this may plausibly correspond to an earlier M170 response to the prime, and a later M170 response to the target, since the target is presented 33.3 ms after the prime. In single word reading studies, the M170 response peaks at ~150 ms post-stimulus onset (Lewis et al., 2011), thus suggesting that there is a slightly later M170 response in a masked priming paradigm. The latency of the second M170 peak is roughly consistent across the conditions of interest: 172 ms for the identity condition, 167 ms for the regular condition, 169 ms for the irregular condition, and 189 ms for the pseudo-irregular condition.

Visual inspection of the related and unrelated mean amplitudes (Figure 2C) reveals an earlier (pre-M170) uncorrected priming effect in the fusiform ROI at ~90–100 ms, suggesting a possible M100 effect of masked priming. The M100 is an evoked response reflecting visual processing of the stimuli, associated with the Type I response from Tarkiainen et al. (1999).

#### M100 analysis

Given the possibility of an early form repetition effect of masked priming, we performed an M100 analysis of the mean amplitude of fusiform activity in the related vs. unrelated PrimeType conditions, collapsed across all 4 classes (identity, regular, irregular, and pseudo-irregular). When corrected for a 50 ms window centered at the typical peak of the M100 response (i.e., 100 ms), the priming effect was not significant (*p* = 0.11 for the cluster at 85–95 ms, MC-corrected for 75–125 ms). However, if we restrict our analysis to the two conditions with the greatest amount of form overlap, namely the identity and regular conditions, then there is a very significant effect (*p* = 0.003 for the cluster at 77–119 ms, MC-corrected for 75–125 ms; Figure 2D).

#### M170 analysis: anatomical fusiform ROI

When we compare the mean amplitude of the fusiform activity in the related vs. unrelated PrimeType conditions, collapsing across the identity, regular, irregular, and pseudo-irregular classes, we find a main effect of PrimeType during the M170 time window, peaking at 180 ms post-target onset (Figure 2C). When corrected for a 50 ms window centered at the peak of the M170 (i.e., 171 ms), the priming effect is significant (*p* = 0.011 for the cluster at 170–195 ms, MC-corrected for 146–196 ms). The direction of the priming effect is such that neural activity in the fusiform ROI is *higher* (i.e., more positive) in the related prime condition.

#### M170 analysis: functional ROI

Our fROI, defined on the basis of the facilitatory identity and regular priming effect, was localized to the middle-to-anterior part of the fusiform and inferior temporal regions (Figure 3A). Visual inspection of the time course of the average activity within this fROI reveals an evoked response moving gradually in the negative (i.e., downward) direction, starting at ~100 ms post-target onset (Figure 3B), consistent with the anterior negative evoked response seen in the temporal lobe for the grand-averaged whole-brain data. When corrected for a 50 ms window centered at the peak of the identity and regular priming effect (i.e., 183 ms), there is a significant effect of PrimeType for the irregular condition (*p* = 0.017 for the cluster at 158–183 ms, MC-corrected for 158–208 ms; Figure 3C), but no effect for the pseudo-irregular condition (Figure 3D). Unlike the M170 anatomical ROI analysis, the direction of the priming effect within the fROI is such that neural activity in the fROI is *reduced* (i.e., less negative) in the related prime condition.

#### M350/N400m analysis: middle temporal ROI

**Figure 4:**
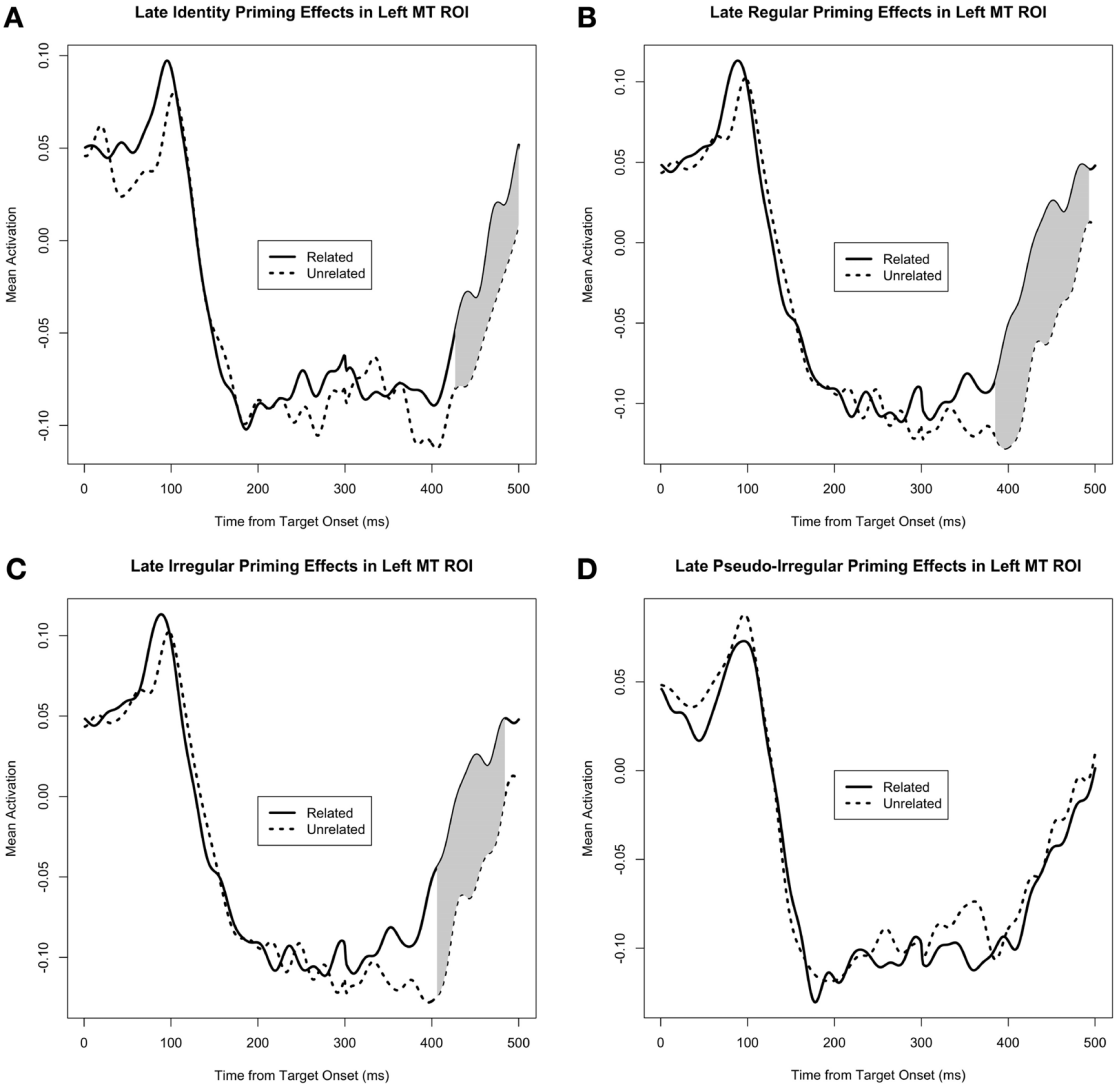
**Mean activity in the middle temporal ROI, separated by PrimeType, and shown for each of the 4 conditions separately: (A) Identity, **(B)** Regular, **(C)** Irregular, and **(D)** Pseudo-irregular**. Significant M350/N400m priming effects are shaded in gray; note that other time windows were not tested for significance.

As can be seen in Figure 4, there is sustained negative activity (i.e., downward with respect to the head) in the middle temporal ROI at ~200–400 ms. Our M350/N400m priming analysis reveals a clear pattern of facilitatory priming effects in this region (i.e., less negative activity for the related PrimeType condition). When corrected for the general late time window 300–500 ms, the priming effects for the identity (*p* = 0.002 for the cluster at 427–500 ms, MC-corrected for 300–500 ms), regular (*p* < 0.0001 for the cluster at 385–493 ms, MC-corrected for 300–500 ms), and irregular (*p* = 0.003 for the cluster at 406–484 ms, MC-corrected for 300–500 ms) priming manipulations were each highly significant on their own, while the pseudo-irregular condition showed no effect (Figure 4). Consistent with this pattern of results, there was a significant interaction of PrimeType and Pseudo-irregularity (*p* = 0.029 for the cluster at 405–439 ms, MC-corrected for 300–500 ms), when comparing only the irregulars and pseudo-irregulars.

### Albrightscore results

The mean AlbrightScore of the irregular verbs was 0.514 (± 0.228), in contrast to the regular verbs, whose mean AlbrightScore was 0.975 (± 0.025); this disparity is due to the fact that the regular rules are always more supported than the irregular rules for past tense formation, given the overwhelming number of regular verbs. Given the tight clustering of the regular AlbrightScore values at close to the maximum value (i.e., 1), we refrained from analyzing them further.

#### Albrightscore behavioral results

First, we tested the effect of AlbrightScore on the degree of RT priming for the irregular verbs. Since AlbrightScore and surface frequency are correlated (*r* = 0.29, *p* < 0.0001), we orthogonalized AlbrightScore with respect to surface frequency (AlbrightScoreO). The effect of the interaction of AlbrightScoreO and PrimeType on RT was not significant for the irregulars (χ^2^ = 1.06, *p* = 0.30).

#### Albrightscore MEG results

**Figure 5:**
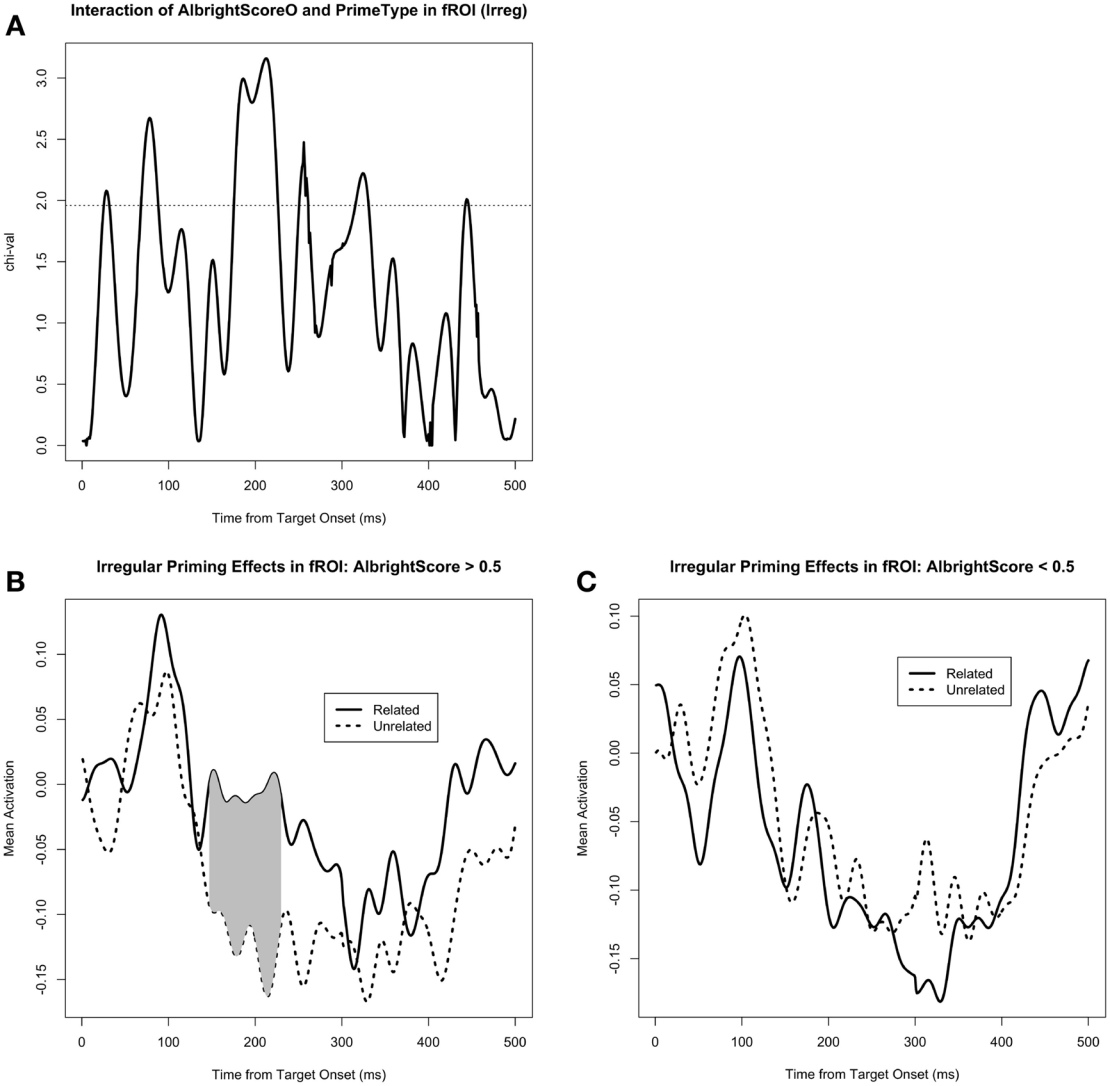
**(A)** The effect of the interaction of AlbrightScoreO and PrimeType on mean activity in the left fusiform/IT functional ROI (fROI), for the irregular condition. The dotted line represents significance at *p* = 0.05 (uncorrected). **(B)** Mean activity in the fROI, separated by PrimeType, for irregular verbs with high AlbrightScore (>0.5). The significant M170 priming effect is shaded in gray; note that other time windows were not tested for significance. **(C)** Mean activity in the fROI, separated by PrimeType, for irregular verbs with low AlbrightScore (<0.5).

We also tested the effect of AlbrightScore on the degree of M170 priming for the irregular verbs (Figure 5). Given the fact that the irregular priming effect was in the expected direction only for the functional ROI analysis, we used that same analysis to look at the effect of AlbrightScore. When corrected for a 50 ms window centered at the peak of the identity and regular priming effect (i.e., 183 ms), the interaction of AlbrightScoreO and PrimeType had a significant effect on activity within the fROI for the irregular condition (*p* = 0.004 for the cluster at 176–208 ms, MC-corrected for 158–208 ms; Figure 5A). When we divide the data into two bins, high AlbrightScore (defined as >0.5) and low AlbrightScore (defined as <0.5), we see a striking disparity: after correction for a 50 ms window centered at the peak of the identity and regular priming effect (i.e., 183 ms), there is a very significant priming effect for the irregulars with high AlbrightScore (*p* = 0.0009 for the cluster at 158–208 ms, MC-corrected for 158–208 ms; Figure 5B), and no effect for the irregulars with low AlbrightScore (Figure 5C).

## Discussion

Our behavioral analysis confirmed that masked presentation of primes significantly facilitated RTs for lexical decision on their targets, in the identity, regular, and irregular conditions, with near-significant facilitation for pseudo-irregulars. This partially confirms the findings of Crepaldi et al. (2010) of RT facilitation due to masked irregular morphological priming, with the caveat that they did not find any hint of priming for the pseudo-irregular condition.

Our MEG analysis confirmed that there is indeed an M170 masked priming effect in the left fusiform gyrus, which is earlier than the effects previously found in MEG studies of masked priming (Monahan et al., 2008; Lehtonen et al., 2011). Interestingly, the direction of the M170 priming effect was such that fusiform activity was greater in the related prime condition than in the unrelated prime condition. Since the direction of this effect is counterintuitive, and we observed that there are actually two, potentially confusable, response components within the same fusiform ROI, we decided to conduct a functional ROI (fROI) analysis as well. The fROI, localized to the middle-to-anterior portion of the fusiform and inferior temporal regions, displayed a significant morphological priming effect for the irregular verbs, and this effect was in the expected facilitatory direction. The presence of an early masked priming effect for irregular verbs suggests that they are decomposed into their stems for lexical access, despite the fact that, unlike regular verbs, they do not necessarily contain their stems in an orthographic sense. Our masked priming results thus provide additional evidence for the single mechanism theory of the English past tense (Stockall and Marantz, 2006), as opposed to the dual mechanism theory (Pinker and Prince, 1988), which would predict early decomposition effects only for regular verbs.

There is an even earlier priming effect for the identity and regular conditions, during the time window of the M100, which was not entirely expected given that the primes and targets were presented in distinct cases. However, there is a precedent in the literature for this type of abstract letter priming: Pylkkänen and Okano (2010) found equal amounts of masked repetition priming for primes and targets in distinct Japanese scripts, as well as visual word form frequency effects at the M100 regardless of the particular script that a word was presented in. Finally, we also found a late M350/N400m masked priming effect in the middle temporal ROI, which was highly significant for the identity, regular, and irregular conditions individually, but not for the pseudo-irregular condition. Thus, while the pseudo-irregular condition displayed a trend toward significance in the behavioral priming analysis, it did not yield similarly significant neural priming effects (in either the M170 or M350/N400m analyses). Given the fact that pseudo-affixed words (e.g., *corner*) do indeed prime their pseudo-stems (e.g., *corn*) in a masked priming paradigm (Rastle et al., 2004), as well as the observation that the transition probability from pseudo-stem to pseudo-affix modulates the M170 in single word reading (Lewis et al., 2011), the failure to obtain clear verification of a pseudo-*irregular* priming effect is surprising. One possibility is that the pseudo-irregular behavioral priming trend is driven by a post-decision process, which may be localized to brain regions outside of left temporal cortex. Additionally, it is possible that the lack of pseudo-irregular MEG priming effects is due to an issue related to the AlbrightScore of the pseudo-irregular pairs, as will be discussed further below.

Our AlbrightScore analysis provides additional evidence supporting the single mechanism account of the past tense. While the behavioral findings were not conclusive, we did find a significant effect of AlbrightScore on the level of priming in the functional ROI for irregular verbs, during the rough time window of the M170 (i.e., 150–250 ms). These results show that the masked morphological priming effect for the irregular verbs only arises because of the high AlbrightScore items; the low AlbrightScore irregulars display no priming effects within the fROI. This confirms the predictions of the single mechanism, form-based, account, in which the irregular past tense forms that are more rule-like (i.e., receive greater support from the general rule structure of how past-tense inflections are computed within English) might be expected to prime their stems to a significantly greater degree than the more exceptional (i.e., less supported) irregulars would, for their respective stems (cf. Stockall and Marantz, 2006, who found different M350 and RT priming effects for high overlap irregular verb-stem pairs, such as *gave*-*give*, and low overlap pairs, such as *taught*-*teach*, with an overt, or unmasked, priming paradigm, and Kielar et al., 2008, who found that *–t* affixed irregular past tense forms prime their stems as effectively as regulars, while *-∅* affixed past tense forms do not, in a masked priming paradigm). Since the masked irregular morphological priming effect seemed to be concentrated at the high end of the AlbrightScore measure for the irregular verbs, it is possible that this fact explains the failure to obtain a significant level of pseudo-irregular priming in the MEG analysis: if the pseudo-irregular condition were analyzed within the high end of an AlbrightScore measure appropriately tailored for those items^7^, we might then observe a significant neural priming effect within those higher AlbrightScore (i.e., more rule-like) pseudo-irregulars.

In summary, the M170 masked morphological priming effect for irregular verbs, as well as the effect of AlbrightScore on the priming effect, suggests that processing of irregular verbs involves application of rules of the sort that generative linguistics predict would be used to map between stems and their past tense forms.

### Conflict of interest statement

The authors declare that the research was conducted in the absence of any commercial or financial relationships that could be construed as a potential conflict of interest.

## Footnotes

^1^While the past tense verb generation models from Albright and Hayes (2003) and Stockall and Marantz (2006) were similar, there was a significant difference between them: Stockall and Marantz (2006) argued that past tense verbs are generated via affixation to a stem, followed by a phonological readjustment to the stem when followed by that affix, while Albright and Hayes (2003) argued that past tense verbs are generated directly via a phonological rule applied to the features of the stem. This distinction will not be explicitly tested in the present study. It is also important to note that Albright and Hayes (2003) were offering an analysis of verb learning, and their predictions regarding online processing of familiar verbs are thus unclear.

^2^We also restricted ourselves to irregular verbs, both as the items in the irregular condition, and as the basis for generating the pseudo-irregulars. Crepaldi et al. (2010) included irregular plural nouns and pseudo-irregulars based on these patterns (e.g., *mice - mouse and spice - spouse*) in their experiments. The set of irregular nouns is much smaller than the set of irregular verbs (i.e., less than 20 irregular nouns vs. at least 150 irregular verbs), and the islands of sub-regularity are consequently much smaller. See Yang (2002) for extensive discussion of the limits of productive rule learning given small sample sizes.

^3^Rastle et al. (2000) did not find a significant masked priming effect for orthographic relatedness (electrode-ELECT) at an SOA of 43 ms, and the effect tended toward inhibition at longer SOAs. Davis and Lupker (2006) noted that most masked priming experiments with word primes reported inhibitory effects of orthographic relatedness, and that the facilitatory effects with word primes noted in some experiments were likely due to the greater length of their stimuli (8–9 letters), which would tend to boost the facilitation of the target (as a result of the larger amount of letter overlap), relative to the inhibition produced by activating its lexical competitors. With word lengths closer to those used in the present experiment (4–5 letters), the inhibitory effects of form overlap are predicted to outweigh the facilitation of the target. Additionally, Rastle et al. (2000) found no masked priming effect for semantic relatedness (cello-VIOLIN) at an SOA of 43 ms, though the effect tended toward significance at longer SOAs.

^4^MNE provides the user with a choice of several orientation constraints for the source estimates: free, fixed, or loose (with respect to the cortical surface normal). Under the latter two orientation constraints, the sign of the resulting source estimates indicates the directionality with respect to the cortical surface: positive indicates a current directed outward from the cortex, and negative indicates a current directed inward toward the cortex. For our choice of free orientation, the sign of the source estimates has a different meaning: positive indicates a current directed upward, and negative indicates a current directed downward, and the coordinate space used to determine these directions is one defined by the head (i.e., MNE's “MEG head coordinate frame,” rather than the cortical surface). Thus, the sign of the data is not meant to indicate the sign of the current, but rather the directionality within this particular coordinate space.

^5^We chose the larger anterior patch (Figure 3A) as the fROI, rather than the (uncorrected) posterior effect, since the anterior effect was stronger, more widespread, and most importantly, in the expected direction (i.e., facilitatory, with a larger magnitude of negative activity in the unrelated condition).

^6^No degrees of freedom are provided for the *t*-values generated by the linear mixed effects models; due to the large number of observations, the *t-distribution* effectively converges to the standard normal distribution (Baayen et al., 2008: Note 1).

^7^It is not obvious how to compute AlbrightScore values for the pseudo-irregular items, since the morphophonological rules in the learning algorithm of Albright and Hayes (2003) take into account the onsets of the words. We would instead need a measure that reflects the similarity of the pseudo-irregular items to the irregular items, in terms of the inflectional patterns of the rhymes (without regard to the onsets).

## Appendix

List of targets, related primes, and unrelated primes.

**Table:**

**Condition**	**Target**	**Related prime**	**Unrelated prime**
Identity	ISLAND	island	proper
	HOSTILE	hostile	grammar
	AWKWARD	awkward	combine
	TREE	tree	busy
	DARE	dare	will
	HEAR	hear	loss
	PROVEN	proven	shabby
	TRUCK	truck	linen
	SHARK	shark	jolly
	TRIUMPH	triumph	advance
	WHITE	white	major
	GOVERN	govern	splash
	SQUAD	squad	crook
	TURBINE	turbine	compass
	CREST	crest	blink
	SIEGE	siege	haunt
	ENVY	envy	axis
	RELISH	relish	canopy
	REPORT	report	animal
	HIRE	hire	tank
	UNION	union	staff
	SPHERE	sphere	button
	RECOVER	recover	publish
	INVENT	invent	borrow
	MONEY	money	start
	BEACH	beach	pound
	FRANK	frank	scoop
	VELVET	velvet	brandy
	DESCEND	descend	buffalo
	EXCESS	excess	virgin
	SACRED	sacred	follow
	PINCH	pinch	meter
	SAKE	sake	wind
	BADGE	badge	imply
	UTILITY	utility	obscene
	REGULAR	regular	mission
	ENGAGE	engage	circus
	PLASTER	plaster	cunning
	SUBSIDY	subsidy	canteen
	SURE	sure	main
	EMPLOY	employ	finish
	BOOTH	booth	decay
	ANGLE	angle	brush
	FRESH	fresh	adult
	COLLEGE	college	husband
	COMFORT	comfort	display
	SUPREME	supreme	contain
	BAMBOO	bamboo	clergy
	CONSENT	consent	library
	OCEAN	ocean	rigid
Regular	RUSH	rushed	ballet
	LOCK	locked	butter
	TALK	talked	decide
	GLOW	glowed	dipped
	KILL	killed	sounds
	REST	rested	hiding
	DASH	dashed	wicker
	LOOK	looked	having
	STAY	stayed	finger
	CHASE	chased	mining
	PASS	passed	winter
	SOUND	sounded	charter
	PUNCH	punched	boasted
	KISS	kissed	repeat
	JUMP	jumped	leaned
	PUSH	pushed	marked
	COOK	cooked	washed
	TURN	turned	seemed
	LIFT	lifted	sudden
	WAIT	waited	humble
	FORM	formed	leaves
	EARN	earned	smooth
	SHOW	showed	middle
	KICK	kicked	begged
	ARM	armed	owned
	TRAIN	trained	couples
	HELP	helped	nation
	WANT	wanted	forces
	FOLD	folded	richer
	GAIN	gained	settle
	WARN	warned	listed
	LAST	lasted	riding
	WISH	wished	regard
	MAIL	mailed	spores
	HEAT	heated	wounds
	WATCH	watched	fingers
	CHEW	chewed	mortar
	CROSS	crossed	meaning
	MIX	mixed	teach
	MISS	missed	pocket
	PULL	pulled	states
	TOUCH	touched	wedding
	CALL	called	pounds
	FILL	filled	master
	WORK	worked	played
	PICK	picked	shared
	REACH	reached	knowing
	FAIL	failed	tucked
	CHECK	checked	artists
	PACK	packed	stores
Irregular	BEAR	bore	pink
	BIND	bound	shape
	BLEED	bled	hawk
	BLOW	blew	pipe
	BREAK	broke	south
	DIG	dug	rub
	DIVE	dove	tart
	DRAW	drew	golf
	DWELL	dwelt	arson
	EAT	ate	sum
	FIND	found	state
	FLEE	fled	pray
	FLY	flew	chin
	GET	got	did
	HANG	hung	trip
	HIDE	hid	mob
	KNEEL	knelt	ghost
	LAY	laid	mood
	MEET	met	job
	PAY	paid	note
	RING	rang	luck
	RUN	ran	hot
	SELL	sold	wing
	SHEAR	shore	bully
	SHINE	shone	tummy
	SHOOT	shot	pain
	SING	sang	fame
	SINK	sank	wary
	SIT	sat	bad
	SLAY	slew	pout
	SLIDE	slid	brow
	SPEAK	spoke	blood
	SPIN	spun	comb
	SPIT	spat	deer
	STAND	stood	group
	STEAL	stole	brink
	STICK	stuck	plane
	STRIKE	struck	annual
	SWEAR	swore	click
	SWIM	swam	toll
	TAKE	took	food
	TEAR	tore	mill
	THINK	thought	because
	WEAVE	wove	shin
	WAKE	woke	tool
	SEND	sent	rock
	WIN	won	led
	SPEND	spent	chair
	FEED	fed	aim
	SMELL	smelt	civic
Pseudo-irregular	PEAR	pore	slow
	HIND	hound	slate
	REED	red	law
	NOW	new	sea
	BLEAK	bloke	troop
	RIG	rug	cab
	LIVE	love	hard
	SAW	sew	hug
	FELL	felt	soon
	MEAT	mate	hull
	MIND	mound	hatch
	BEE	bed	cut
	CRY	crew	poet
	PET	pot	sad
	SLANG	slung	chore
	BIDE	bid	log
	PEEL	pelt	rift
	PLAY	plaid	tweed
	BEET	bet	gap
	RAY	raid	vice
	CLING	clang	trump
	BUN	ban	mat
	BELL	bold	sack
	SEAR	sore	bull
	LINE	lone	boot
	LOOT	lot	big
	PING	pang	musk
	RINK	rank	belt
	PIT	pat	bay
	DAY	dew	cue
	RIDE	rid	nab
	PEAK	poke	hush
	SIN	sun	low
	FLIT	flat	hope
	HAND	hood	zest
	REAL	role	must
	SICK	suck	flap
	BIKE	buck	clap
	GEAR	gore	colt
	RIM	ram	bob
	BAKE	book	cold
	FEAR	fore	mink
	DRINK	drought	massage
	GRIEVE	grove	flank
	STAKE	stoke	holly
	TEND	tent	bill
	TIN	ton	jay
	VEND	vent	gash
	WEED	wed	lax
	WELL	welt	toot
